# The role of osmotic stress transcription factor 1 in fishes

**DOI:** 10.1186/s12983-014-0086-5

**Published:** 2014-11-19

**Authors:** William Ka Fai Tse

**Affiliations:** Department of Biology, Hong Kong Baptist University, Kowloon Tong, Hong Kong

**Keywords:** Gill, Osmoregulation, Osmosensing, Osmotic stress

## Abstract

Osmotic stress transcription factor 1 (Ostf1) was first discovered by subtractive hybridization in the gills of Mozambique tilapia (*Oreochromis mossambicus*) transferred from fresh water (FW) to seawater (SW). It is a putative transcriptional regulator and the “early hyperosmotic regulated protein”. In the 2 hours after FW to SW transfer, *ostf1* mRNA levels increase six fold. It is believed that, as a fast-response gene, Ostf1 plays a critical role in fish osmoregulation. Since its discovery, numerous studies have been performed to understand the nature and osmoregulatory mechanism of Ostf1. A decade has passed since the discovery of Ostf1, and it is a good time to summarize our current understanding of this gene. Different fish models have been used to study Ostf1, which is not limited to the traditional euryhaline fishes, such as eels and tilapia. Ostf1 can be found in modern fish models such as medaka and zebrafish. This review covers and summarizes the findings from different fishes, and provides a perspective for future Ostf1 studies.

## Introduction

The capability of animal cells to maintain cell volume and structural dynamics are prerequisite for cellular life. This is particularly important for euryhaline fishes, as they must maintain water and ion homeostasis in their gills during migration. Numerous cellular events occur during osmotic stress, such as changes in the activities of cellular receptors and reorganization of the cellular cytoskeleton architecture [1,2]. Fiol and Kultz proposed the concept of an “osmosensory signal transduction network” in order to summarize cellular events during osmoregulation in fishes. The authors divided the osmoregulatory process into three parts: osmosensors, signal transducers, and effectors. Cellular sensors detect external osmolality changes and stimulate various signaling molecules, which induce effectors to compensate for the osmotic challenge [3]. Researchers have spent decades attempting to understand the underlying osmoregulatory mechanism in fishes. However, due to the complicated factors involved in the process, (for example, how changes in external ion contents or internal hormonal levels affect osmotic responses), it is still unknown which factors or molecules are critical to the process. Using traditional methods, such as microarray, subtractive hybridization, and 2-D gel studies, researchers have identified several potential factors. Osmotic stress transcription factor 1 (Ostf1) is one of these factors and has been investigated since its discovery in Mozambique tilapia (*Oreochromis mossambicus*) in 2005. Ostf1 is an “interesting” molecule for study, as it responds rapidly and specifically to osmotic stress and is not induced by oxidative stress or heat shock [4]. This indicates that it may be involved in regulating the downstream osmoregulatory mechanisms during the early stages of acclimation. In recent years, we have gained a better understanding of the effectors (end-points) during osmotic stress, such as changes in ion transporters and channels. For example, gill ion transporters, such as the Na^+^/K^-^/2Cl^-^ (NKCC) and cystic fibrosis transmembrane conductance regulator (CFTR) Cl^-^ channels are upregulated after seawater acclimation [5]. We also now know that different signaling pathways, such as the myosin light chain kinase (MLCK), focal adhesion kinase (FAK), and mitogen-activated protein kinase (MAPK) pathways, are stimulated by osmotic stress [6–10]. Furthermore, recent morpholino knockdown technology has been applied in physiological studies of zebrafish to provide direct functional evidence of some hormonal receptors such as glucocorticoid receptor and calcium sensing receptor [11,12]. As there have been several reviews [2,13,14] that have summarized the findings of these studies, we will not explore them in detail here. Although knowledge of the osmoregulatory pathway has increased, the molecules that trigger or stimulate effectors (signal transducers) are still not fully understood. The mRNA expression of Ostf1 increases within 6 hours after transfer from fresh water (FW) to sea water (SW), but later returns to the background level. This suggests that it may act as a signal transducer during osmotic stress [4,15]. Researchers have attempted to identify the regulators and direct downstream functions of Ostf1 in various species using various methods. Here, we provide an overview of Ostf1 studies conducted over the last 10 years. We summarize the data, suggest a general working model of Ostf1, and provide suggestions for future Ostf1 studies. To ease reading and understanding, we have consolidated the research data and included only the central idea of each previous study. For detailed experimental set-up or discussion, please refer to the original articles.

### Ostf1 in tilapia

Ostf1 was first identified in Mozambique tilapia and further studies have confirmed that the level of tilapia *ostf1* mRNA expression in gills or prolactin-secreting cells increases when fish are transferred from FW to SW [4,16], or in response to an increase in extracellular osmolality [17]. Another follow-up *in vitro* gill cell study showed that the activation of Ostf1 depends on hypertonicity as the stimulatory signal, and that its activity is related to transient mRNA stability [18]. Furthermore, the relationship between cortisol and Ostf1 was studied due to the high similarity between the transforming growth factor-beta-stimulated clone-22 (TSC22) domain in the Ostf1 and the mammalian glucocorticoid-induced leucine zipper (GILZ). The presence of the TSC22 domain suggested that Ostf1 may be regulated by the seawater-adapting hormone, cortisol. Hormone regulation is critical for acclimation to SW [19,20]. The GILZ domain in Ostf1 suggests that glucocorticoids could stimulate Ostf1. However, in an *in vitro* gill cell culture study by the same group, Ostf1 was not stimulated by the synthetic glucocorticoid receptor agonist dexamethasone (DEX) [18]. This negative result, however, did not dissuade researchers from attempting to understand the relationship between cortisol and Ostf1. In 2010, it was reported that direct intraperitoneal (IP) injection of cortisol into FW tilapia for 3 days increased the level of *ostf1* mRNA expression and the survival rate of fish transferred directly from FW to SW [21]. These results indicate that the tilapia *ostf1* may be responsive to cortisol only in the *in vivo* system.

In addition to Mozambique tilapia, Nile tilapia (*Oreochromis niloticus*) have been used to determine the Ostf1 mechanism. The Mozambique tilapia has a higher osmotic tolerance than Nile tilapia, which led to some species-specific findings regarding the role of Ostf1. For example, although *ostf1* mRNA expression increases in both species after acute post FW to SW transfer, *ostf1* is induced only in the Nile tilapia after brackish water to SW transfer [16]. These results may be explained by the need for *ostf1* to boost osmoregulation in the species (Nile tilapia) with weaker osmotic tolerance. Recently, Yan et al. suggested that Nile tilapia micro RNA 429 (miR429) is responsible for the regulation of *ostf1*. miRNAs are endogenous ~22-nt RNAs that play important regulatory roles by targeting mRNAs for cleavage or translational repression [22]. Through bioinformatics analysis, Yan and his colleagues identified a potential miR429 binding site at the 3′ end of *ostf1*, suggesting that miR429 may act to suppress *ostf1*. Furthermore, an *in vivo* experiment demonstrated that the inhibition of miR429 increases *ostf1* expression, and that the knockdown of miR429 influences plasma osmolality [23]. Although studies of miRNA and fish osmoregulation are limited, Yan et al. were the first to link miRNA to *ostf1*, which has stimulated research on the functions of miRNA in osmoregulation. Recently, reports have shown that miRNA can regulate the ion transport or osmotic response element binding protein in mammals [24,25]. We foresee that miRNA studies in fishes will further uncover the osmoregulatory mechanism in the near future.

### Ostf1 in Japanese eel

Our group has for a number of years used the Japanese eel (*Anguilla japonica*) as a model to study osmoregulation. We have identified different osmoregulatory mechanisms in the model, such as changes in the expression levels of various ion transporters under different osmotic stresses [5,26,27]. In 2008, our group cloned the full length of Ostf1 from eel gills. The sequence has more than 80% homology with tilapia Ostf1 [15]. The two major cell types for osmoregulation in eel gills are the pavement cells (PVCs) and mitochondria-rich cells (MRCs). The expression of *ostf1* mRNA increased after FW to SW transfer in both cells, with a higher induction in MRCs [15]. In an immunohistochemistry staining study of gill cells, Ostf1 was found to be co-localized in the phosphorylated-extracellular signal regulated kinase (p-ERK) MRCs after FW to SW transfer [28]. Hyperosmotic stress activates mitogen-activated protein kinase (MAPK) pathways [29], in which the activation of Ostf1 may be related to the phosphorylation of ERKs in eel gill cells during seawater acclimation. Gill cell culture was used in addition to whole animal studies to unfold the molecular regulation of Ostf1. We have shown that eel *ostf1* does not respond to permeable solutes, such as urea, whereas the addition of a microtubule polymerization inhibitor (colchicine) reduces *ostf1* expression during hyperosmotic stress [15]. The study provided the first indication that Ostf1 might affect cytoskeleton reorganization during osmotic stress. The theory was further supported in a study of medaka that will be described in the next section. Eel gill cell culture experiments also showed that DEX can stimulate *ostf1* mRNA expression via glucocorticoid receptors and that this expression is mediated by the Akt-GSK3β signaling pathway [30].

### Ostf1 in freshwater medaka

Fresh water medaka (*Oryzias latipes*) is a common modern model for biological research. It has shown its value in developmental biology. Medaka is a euryhaline fish, and is also a good model to study osmoregulation [10]. The advantage of using this modern model organism for the study of osmoregulation is its well-known genome. From a genome-wide search of Ostf1-like genes in medaka, there are nine transcripts (splicing forms from three genes) with the TSC22 domain. Four of these transcripts are stimulated within 6 hours of FW to SW transfer. Among these four transcripts, two transcripts of TSC22D3 are highly activated. Their amino acid sequences are highly similar to that of Mozambique tilapia Ostf1 (88.7% and 44.3%). Therefore, we named these transcripts Ostf1 and Ostf1b. The relatively low similarity between TSC22D3-2 (Ostf1b) and tilapia Ostf1 is mainly due to its shortened 5′ end nucleotide sequence (72 fewer amino acids in TSC22D3-2 than in tilapia Ostf1). Nevertheless, because the mRNA expression level of *ostf1b* is greatly stimulated (4–10 fold) during acute hyperosmotic stress (30 mins to 6 hrs post FW to 50% SW transfer), we suggested that medaka Ostf1b is an ortholog of tilapia Ostf1 [10]. The data suggested that the shortened 5′ end is not important for the response to osmotic stress in medaka. Further studies have been performed to understand the role of medaka Ostf1b. The results of these studies suggest that Ostf1b stimulates the JNK pathway via GCK at the post-transcriptional level during hyperosmotic stress. In addition, in *in vitro* human embryonic kidney (HEK293) cell culture studies, inhibition of JNK phosphorylation by the specific inhibitor SP600125 resulted in a decrease in Ostf1b protein levels in ectopic Ostf1b expressed cells. Furthermore, three critical ion transporters and channels in osmoregulation (*AQP1, CFTR,* and *NHE3*) have a direct stimulatory response to Ostf1b (increase in mRNA expression level) in the ectopic expression of Ostf1b in HEK293 cells [10]. Another *in vitro* study has shown that Ostf1b can promote cell migration properties by modulating the epithelial mesenchymal transition (EMT) phenotype in HEK293 cells. In this study, Ostf1b stimulated Rho kinase 1 (ROCK1) and myosin light chain 2 (MLC2) phosphorylation, which resulted in the contraction of myosin rings. Additionally, activation of ROCK1 stimulated the expression of two tight junction proteins, Occludin and Claudin 1, thereby causing the disassembly of tight junctions. These phenotypes stimulated cell migration, which supports the notion of osmotic-stress-induced gill cell migration [31]. These two studies outlined the general signaling flow of Ostf1b and suggested that Ostf1b plays a role in regulating both ion transporters and the cytoskeleton during osmotic stress.

### Ostf1 in zebrafish

Zebrafish (*Danio rerio*) is a FW fish that does not tolerate hyperosmotic stress. Until recently, there were no studies of Ostf1 in stenohaline fishes. Zebrafish, as a very common modern model, is widely used in the study of developmental biology. Recently, it has become an excellent drug and disease model [32,33]. Researchers have also taken advantage of the ease of genetic modification in this animal to study osmoregulation [34]. Through a general search for Ostf1 in its genome database, nine transcripts with the TSC22 domain were found. Protein alignment indicates that of these transcripts, Tsc22d3 is the most similar to tilapia Ostf1 (43.3%), and medaka Ostf1b (82.9%). Unlike medaka Ostf1, there is no splicing form of zebrafish Tsc22d3. Therefore, zebrafish Tsc22d3 was suggested to be Ostf1. Zebrafish Tsc22d3 contains 143 amino acids, which is similar to medaka Ostf1b. Studies of adult zebrafish gills and zebrafish embryos indicated that Ostf1 (Tsc22d3) was not responsive to hyperosmotic stress in these animals. In addition, knockdown of Ostf1 in zebrafish embryos does not affect any mRNA expression of ionocyte markers (*atp1b1b, atp6v1a,* and *foxi3a*), which indicates that Ostf1 does not directly participate in osmoregulation or the development of osmoregulatory cells (ionocytes). Although Ostf1 is not responsive to osmotic stress in zebrafish, the knockdown of Ostf1 in embryos led to the discovery of its important roles in early embryogenesis. Ostf1 is a ventralized gene that is important for the growth of the ventral region of fishes. Ostf1 can stimulate the transcription of bone morphogenetic protein 4 (*bmp4*), resulting in a positive effect on the Bmp signaling pathway [35]. The results provided knowledge in addition to the general osmoregulatory function of Ostf1.

### Ostf1 in other fishes

Although there are limited studies of Ostf1 in other fishes, it has been studied in black porgy (*Acanthopagrus schlegeli*). In this study, unlike in the other studies mentioned above, fish were transferred from SW to FW. Interestingly, Ostf1 was stimulated in gills after SW to FW transfer. This study was the first to show that Ostf1 is responsive to hypo-osmotic stress [36]. Using a microarray approach to study Atlantic salmon (*Salmo salar*), researchers found that TSC22D3 was downregulated in the liver, head kidney, and muscle after infection by a bacterium, *Piscirickettsia salmonis* [37]. The study further demonstrated that, in addition to its role as an osmoregulatory gene, Ostf1 may have other, unknown functions.

### Conclusions and perspectives

**Figure 1:**
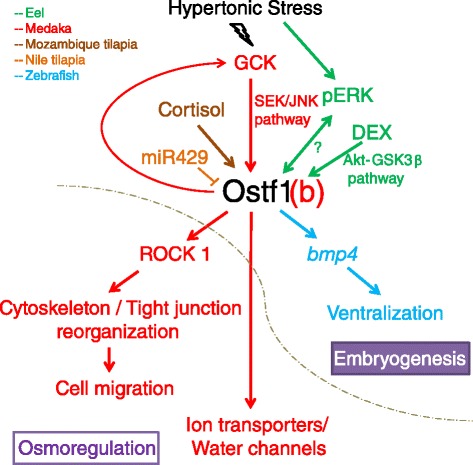
**Proposed model of Ostf1 in fishes.** The model combines all the findings from various fishes. Different colors represent different fishes (green for eel, red for medaka, brown for Mozambique tilapia, orange for Nile tilapia, and blue for zebrafish). It should be noted that the model might not be fitted for all fishes; however, it suggests the general signaling flow of Ostf1 in fishes from its regulators to downstream functions. Different molecules or stressors could regulate the expression of Ostf1. Hyperosmotic stress is one such stressor. In medaka, osmotic stress activates the SEK/JNK pathway to stimulate Ostf1b [21]. In addition, cortisol injection into Mozambique tilapia stimulates the expression of Ostf1 [11]. In an eel gill cell culture model, DEX was shown to induce Ostf1 via the Akt-GSK3β pathway [20]. Furthermore, eel gill immunohistochemical staining has shown the activation of p-ERK and Ostf1 after fresh water to seawater transfer. p-ERK and Ostf1 co-expressed at the same time and further experiments are required in order to determine whether pERK stimulates Ostf1, or vice versa [18]. The final regulator in this model is miR429 in Nile tilapia. Similar to other miRNAs, miR429 may inhibit the expression of *ostf1* mRNA [12]. The induction of Ostf1 leads to different downstream functions. In euryhaline fresh water medaka, Ostf1b may further activate the ROCK pathway for cytoskeleton reorganization, and cell migration [22]. Furthermore, at the same time, it may directly stimulate the mRNA expression of different ion transporters or channels to maintain water and ion homeostasis [21]. These osmoregulatory processes have been shown to be related to the Ostf1b. In the stenohaline zebrafish, Ostf1 functions as a ventralizing gene that plays critical dorsal-ventral roles during early embryogenesis [26]. To summarize, Ostf1 has different functions in fishes, ranging from regulating osmotic responses (osmoregulation) to playing critical roles in early development (embryogenesis).

Since the discovery of Ostf1 in tilapia, Ostf1 homologs have been identified in various different fishes. Most of the identified Ostf1 are responsive to hyperosmotic stress. Classical models, like tilapia and eel, have been used to study the gene. However, due to limited information about their genomes and the difficulties of performing molecular functional *in vivo* experiments using these models, researchers have used modern model organisms to unfold the underlying mechanism and functions of Ostf1. By combining *in vivo* and *in vitro* studies, together with the genetic modification technology, we now have some idea as to how Ostf1 is regulated. Figure 1 summarizes the findings of Ostf1 studies in different fishes. We now have a basic understanding of the regulators and downstream functions of Ostf1; however, there are still questions that remain unanswered:

Ostf1 function:

1. Is it true that Ostf1 does not have osmoregulatory functions in all FW fishes? If so, is it involved in dorsoventral patterning, as in zebrafish?
2. Is Ostf1 essential in euryhaline fishes? Without Ostf1, will fish tolerance to osmotic stress be reduced? If Ostf1 is overexpressed in FW fishes, will they survive FW to SW transfer? Is it true that FW fishes cannot resist hyperosmotic stress because of the “non-osmotic function of Ostf1”?
3. What is the function of Ostf1 in other non-osmoregulatory organs? Does it have any specific roles in different organs?
4. Is there any relationship between the localization of Ostf1 in gill MRCs and osmoregulatory functions?
5. Does medaka Ostf1b have the same ventralizing function in FW medaka during early development as Ostf1 in zebrafish? As medaka and zebrafish are two related teleost species with 115–200 Myr of independent evolution, why does Ostf1 have different osmoregulatory functions or additional developmental functions in these two species?

Molecular regulation:

1. Is Ostf1 a real transcription factor? To fully understand the molecular regulation of Ostf1, it is essential to identify its promoter region in fishes, particularly in non-model organisms like eels and tilapia.
2. Are there any common signaling pathways for Ostf1? Phosphorylation in MAPKs is activated in euryhaline fishes, such as eel and medaka, during osmotic stress, whereas in freshwater zebrafish, the BMP pathway is involved during early development.
3. What is the molecule that triggers the induction of Ostf1? Because Ostf1 is stimulated during acute osmotic stress, it is reasonable to assume that the concentrations of certain specific ions or osmolytes in seawater are responsible for Ostf1 induction. What are these ions or osmolytes?

To answer these questions, we suggest the combined use of transgenic and next-generation sequencing (NGS) approaches in modern model organisms, such as zebrafish or medaka. To understand the expressional changes of Ostf1 during development or osmotic stress, a transgenic line should be generated. A line with Ostf1-driven green fluorescent protein (GFP) would be very useful. This line could be used to trace Ostf1-expressing cells during development or transfer-related osmotic stress. Genetic modification of Ostf1, such as the knockout of Ostf1 by transcription activator-like effector nuclease (TALEN) or Clustered Regularly Interspaced Short Palindromic Repeats (CRISPR), could be used to determine the effects of modifications on osmotic tolerance. A deeper understanding of Ostf1 could be gained by using NGS (RNA seq) studies to compare the deregulatory genes and networks of control and Ostf1 knockout fish.

We hope that this article has provided some insights and ideas to different researchers, and we look forward to their contributions to our understanding of Ostf1 in the near future.

## Abbreviations

BMP: Bone morphogenetic protein
CRISPR: Clustered regularly interspaced short palindromic repeats
DEX: Dexamethasone
EMT: Epithelial mesenchymal transition
FAK: Focal adhension kinase
FW: Fresh water
GILZ: Glucocorticoid-induced leucine zipper
GFP: Green fluorescent protein
HEK293: Human embryonic kidney cells
IP: Intraperitoneally
MRCs: Mitochondria-rich cells
MAPKs: Mitogen-activated protein kinases
MLC2: Myosin light chain 2
NGS: Next generation sequencing
Ostf1: Osmotic stress transcription factor 1
PVCs: Pavement cells
p-ERK: Phosphorylated-extracellular signal regulated kinase
ROCK1: Rho kinase 1
SW: Seawater
TALEN: Transcription activator-like effector nuclease
TSC22: Transforming growth factor-beta-stimulated clone-22
